# Molecular and cellular functions of the FANCJ DNA helicase defective in cancer and in Fanconi anemia

**DOI:** 10.3389/fgene.2014.00372

**Published:** 2014-10-21

**Authors:** Robert M. Brosh, Sharon B. Cantor

**Affiliations:** ^1^Laboratory of Molecular Gerontology, National Institute on Aging, National Institutes of HealthBaltimore, MD, USA; ^2^Department of Cancer Biology, University of Massachusetts Medical School – UMASS Memorial Cancer CenterWorcester, MA, USA

**Keywords:** FANCJ, helicase, DNA repair, replication, Fanconi anemia, cancer, genomic stability, G-quadruplex

## Abstract

The FANCJ DNA helicase is mutated in hereditary breast and ovarian cancer as well as the progressive bone marrow failure disorder Fanconi anemia (FA). FANCJ is linked to cancer suppression and DNA double strand break repair through its direct interaction with the hereditary breast cancer associated gene product, BRCA1. FANCJ also operates in the FA pathway of interstrand cross-link repair and contributes to homologous recombination. FANCJ collaborates with a number of DNA metabolizing proteins implicated in DNA damage detection and repair, and plays an important role in cell cycle checkpoint control. In addition to its role in the classical FA pathway, FANCJ is believed to have other functions that are centered on alleviating replication stress. FANCJ resolves G-quadruplex (G4) DNA structures that are known to affect cellular replication and transcription, and potentially play a role in the preservation and functionality of chromosomal structures such as telomeres. Recent studies suggest that FANCJ helps to maintain chromatin structure and preserve epigenetic stability by facilitating smooth progression of the replication fork when it encounters DNA damage or an alternate DNA structure such as a G4. Ongoing studies suggest a prominent but still not well-understood role of FANCJ in transcriptional regulation, chromosomal structure and function, and DNA damage repair to maintain genomic stability. This review will synthesize our current understanding of the molecular and cellular functions of FANCJ that are critical for chromosomal integrity.

## DISCOVERY OF BACH1/BRIP1/FANCJ AND ITS EMERGENCE AS A PROMINENT PLAYER IN HUMAN GENETIC DISEASE AND TUMOR SUPPRESSION

FANCJ (originally named BRCA1 interacting C-terminal helicase (BACH1) or BRCA1 interacting helicase (BRIP1) was first discovered by its physical interaction with BRCA1, a known tumor suppressor and mediator of double strand break (DSB) repair (Cantor et al., 2001). Consistent with FANCJ’s association with BRCA1, FANCJ-deficient cells are sensitive to DNA cross-linking agents (Bridge et al., 2005; Levitus et al., 2005; Litman et al., 2005) and mildly sensitive to ionizing radiation (IR; Peng et al., 2006), and display a defect in homologous recombination (HR) repair of DSBs (Litman et al., 2005). The first clinical evidence for the importance of FANCJ was the identification of germ line sequence changes in FANCJ that were associated with early breast cancer in two individuals that displayed normal genotypes for BRCA1 and BRCA2 (Cantor et al., 2001). Subsequent studies solidified the causal relationship of FANCJ mutations as low penetrance breast and ovarian cancer alleles (Seal et al., 2006; Rafnar et al., 2011; for review, see Cantor and Guillemette, 2011).

**FIGURE 1:**
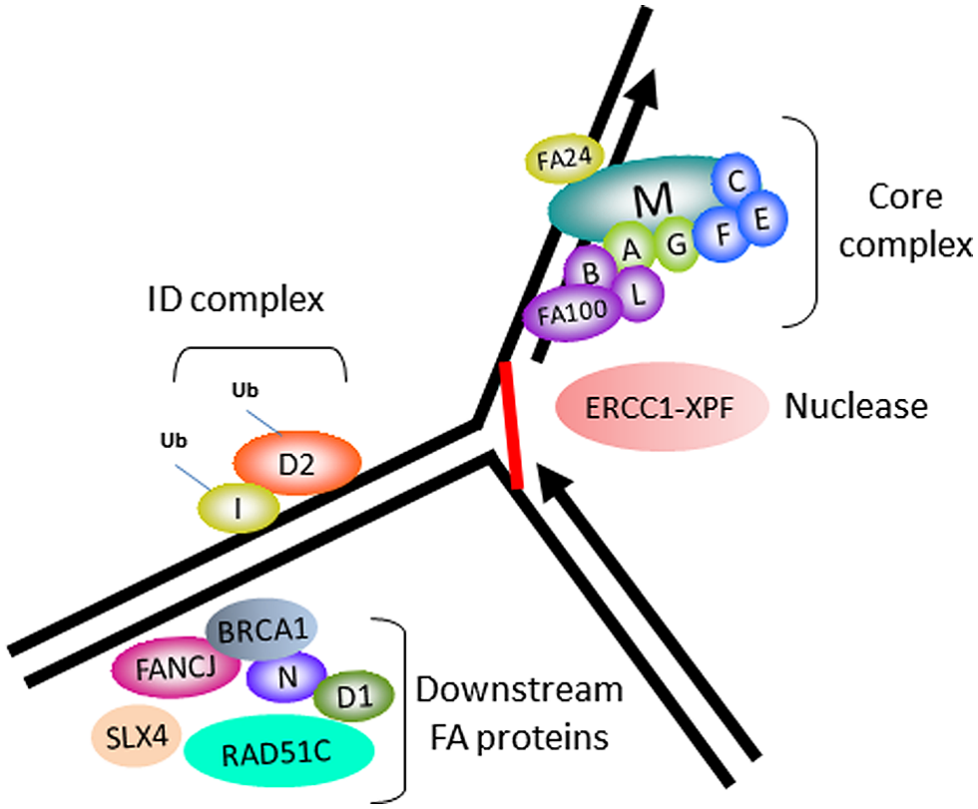
**Fanconi anemia (FA) proteins implicated in interstrand cross-link (ICL) repair.** Cartoon schematic of the FA proteins implicated in the ICL repair pathway. The integrity of the core complex is required for FANCD2/FANCI monoubiquitination, a key activation step of the FA pathway. ERCC1-XPF is the nuclease implicated in ICL processing. FANCJ and certain other FA proteins are not required for FANCD2/FANCI monoubiquitination, and are therefore implicated in downstream events of the FA pathway, such as homologous recombination (HR) repair of the processed ICL.

In accord with its role as a tumor suppressor, FANCJ was identified as the gene mutated in the J complementation group of Fanconi anemia (FA), a rare disorder characterized by progressive bone marrow failure, skeletal abnormalities, and cancer (Levitus et al., 2005; Levran et al., 2005; Litman et al., 2005). Currently, there are 16 FA complementation groups. The corresponding genes encode proteins implicated in a complex pathway of interstrand cross-link (ICL) repair that corrects damage when the two complementary strands of the DNA double helix become covalently linked, a type of lesion that blocks cellular DNA replication and transcription (**Figure 1**). The reader is referred to several recently published excellent reviews on the functions of the FA gene products and the overall workings of the FA pathway and its importance in chromosomal stability (Kee and D’Andrea, 2012; Kottemann and Smogorzewska, 2013; Walden and Deans, 2014). One notable finding is that FANCJ is not required for DNA damage induced FANCD2 monoubiquitination, suggesting that the helicase functions downstream of this key activation step of the FA pathway (Litman et al., 2005). FANCJ likely operates with other downstream BRCA-FA proteins, such as BRCA1, and related factors also classified as tumor suppressors to facilitate recombinational repair (potentially following unhooking of the processed cross-link; **Figure 1**). In addition, it is believed that FANCJ functions in a broader role to suppress replication stress (see subsequent sections).

**FIGURE 2:**
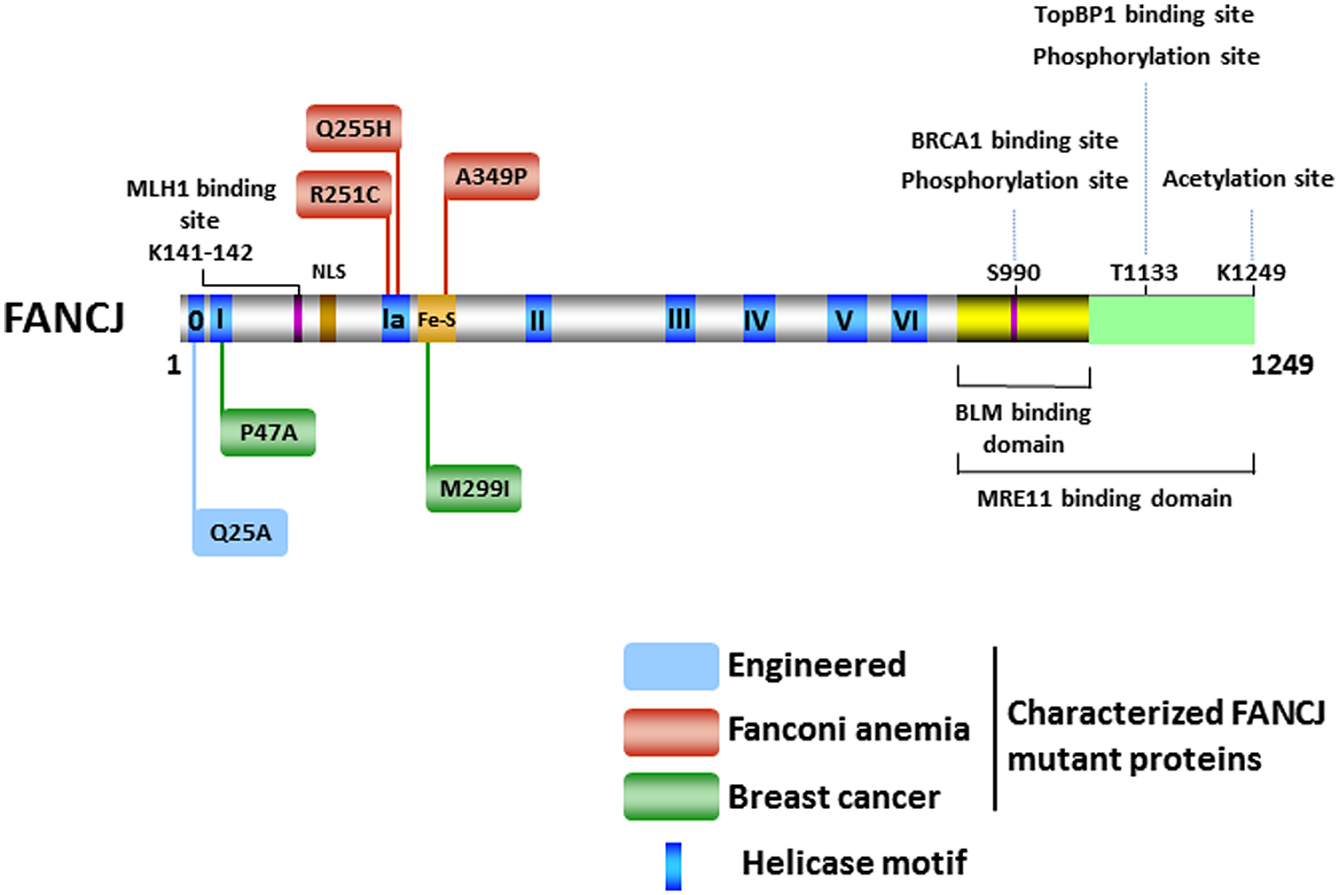
**FANCJ and its catalytic helicase domain, characterized site-directed mutations, sites of post-translational modifications, and protein interactions.** See text for details.

The FANCJ gene encodes a protein of 1,249 amino acids with a conserved ATPase helicase core domain comprised of eight motifs [0 (Q), I, Ia, II, III, IV, V, VI] found in DEAH superfamily two helicases (**Figure 2**). A signature motif in this FANCJ family of DNA helicases is an Iron–Sulfur (Fe–S) cluster, characterized by four conserved cysteine residues, residing within the helicase core domain (Cantor et al., 2004; Rudolf et al., 2006). Members of the Fe–S helicase cluster family function in preserving the genome, such as XPD and RTEL helicases (Wu et al., 2009). Recently solved crystal structures and biochemical studies of thermophilic species of XPD suggest that the Fe–S cluster functions with an Arch domain that is proposed to be a wedge propelling strand separation. Moreover, two conserved RecA-like domains mediate ATP binding, protein–DNA interactions, and helicase translocation mediated by ATP-induced conformational changes (Rudolf et al., 2006; Fan et al., 2008; Wolski et al., 2008; Kuper et al., 2011). Studies on Fe–S cluster helicases XPD (Mui et al., 2011; Sontz et al., 2012) and DinG (Ren et al., 2009), as well as other DNA processing enzymes (Grodick et al., 2014), have implicated the Fe–S cluster as a redox sensor that facilitates DNA damage detection; however, a formal role of the FANCJ Fe–S cluster in this capacity has not been determined.

## CATALYTIC ACTIVITIES AND DNA SUBSTRATE SPECIFICITY OF FANCJ HELICASE

Biochemical characterization of the purified recombinant FANCJ protein using a classic DNA directionality substrate (Cantor et al., 2004) or oligonucleotide-based partial duplex substrates with single-stranded DNA overhangs of defined polarity (Gupta et al., 2005) determined the 5′–3′ directionality of FANCJ as an ATP-dependent DNA helicase. Biophysical analyses of FANCJ assembly state suggest that the helicase exists in an equilibrium, as a monomer, and dimer in solution. Furthermore, biochemical studies showed that the dimeric form of FANCJ displays maximal catalytic activity as an ATPase and DNA helicase on relatively short forked duplex substrates of 20 base pairs (bp; Wu et al., 2012). FANCJ is limited in its processivity; therefore, it poorly unwinds substrates with duplexes of ∼50 bp or greater. However, on both shorter and longer duplexes its helicase activity is markedly stimulated by the single-stranded DNA binding protein replication protein A (RPA; Gupta et al., 2007).

**FIGURE 3:**
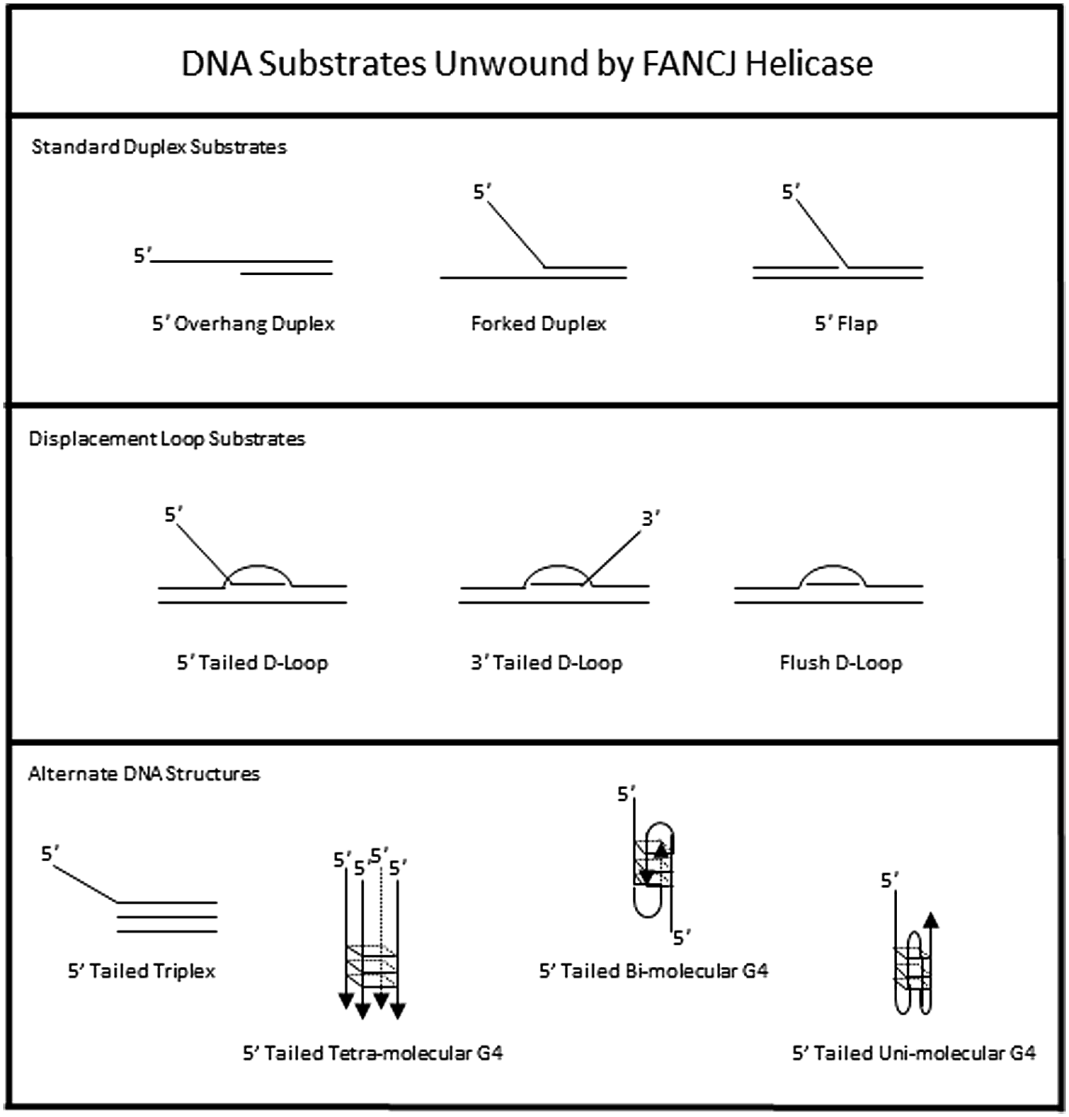
**DNA substrate specificity of FANCJ helicase.** See text for details.

The DNA substrate preference of FANCJ has been studied in some detail (**Figure 3**). A 5′ tail of 15 nucleotides (nt) is required and 35 nt is optimal for FANCJ to catalyze appreciable unwinding of the simple 5′ tailed duplex (Gupta et al., 2005). FANCJ preferentially binds and unwinds forked duplex DNA substrates (Gupta et al., 2005), and is also active on a 5′ flap (but not 3′ flap) substrate (**Figure 3**), consistent with its translocation directionality. Backbone continuity in the pre-existing 5′ single-stranded DNA tail, but not the 3′ single-stranded tail, of the forked duplex substrate within six nt of the single-stranded DNA-double stranded DNA junction is required for FANCJ to initiate unwinding of the adjacent duplex. However, disruption of the sugar phosphate backbone by a polyglycol modification in *either* the translocating or non-translocating strand within the duplex region inhibits FANCJ helicase activity (Gupta et al., 2006). This finding demonstrates that FANCJ senses both strands during the elongation phase of the unwinding reaction. Inhibition of FANCJ helicase by the polyglycol modification in either strand of the duplex substrate can be overcome by increasing the 5′ single-stranded DNA loading tail of the substrate (Gupta et al., 2006), suggesting that loading of multiple FANCJ molecules under multi-turnover conditions drives forward the DNA unwinding reaction even when the helicase encounters a formidable obstacle to progression. Interestingly, FANCJ helicase activity is not inhibited by the presence of abasic sites in either the translocating or non-translocating strands within the duplex region of the forked DNA substrate (Gupta et al., 2006), suggesting that FANCJ’s electrostatic interactions with the sugar phosphate backbone dominate over base-stacking interactions. FANCJ helicase activity is also inhibited in a translocating strand specific manner by an alkyl phosphotriester lesion that introduces a hydrophobic group into the nucleic acid backbone and neutralizes the negatively charged phosphodiester moiety (Suhasini et al., 2012). Presumably, the physical attributes of the alkyltriester damage or its effect on double helical rigidity differentially affect FANCJ unwinding compared to the polyglycol linkage, which inhibited irrespective of the strand. A number of chemical genotoxins cause the formation of phosphotriester adducts which can persist for a long time in genomic DNA (Jones et al., 2010). These lesions and other DNA adducts may exert their mutagenic and carcinogenic effects by inhibiting DNA metabolizing enzymes, including helicases such as FANCJ (Suhasini and Brosh, 2010).

To assess the possibility that FANCJ has a catalytic role in processing an ICL intermediate in a DSB repair pathway, we assessed its activity on a three-stranded displacement loop (D-loop) DNA substrate that represents a key early intermediate of HR repair (Gupta et al., 2005). Following unhooking of an ICL, a D-loop arises after RAD51-mediated strand invasion and base-pairing of a single-stranded DNA overhang formed at a resected DSB. Notably, FANCJ unwinds a D-loop without a 5′ single-stranded DNA tail suggesting that the DNA junctions in the D-loop substrate enable FANCJ to overcome its usually strict requirement for loading on a 5′ single-stranded DNA tail to initiate unwinding (**Figure 3**). However, FANCJ fails to unwind a four-stranded Holliday Junction structure, another key intermediate of HR repair that can lead to cross-over or non-crossover recombinant products (Gupta et al., 2005). Thus, FANCJ may be exquisitely tailored to act upon D-loop intermediates to suppress HR or homeologous recombination. Alternatively, FANCJ may act upon D-loops to enable synthesis-dependent strand annealing, a pathway of DSB repair distinct from the classic Holliday Junction resolution pathway.

In addition to unwinding conventional duplex DNA substrates, FANCJ resolves alternate DNA structures including DNA triplexes (Sommers et al., 2009) or G4 (London et al., 2008; Wu et al., 2008) that form by Hoogsteen hydrogen bonding (**Figure 3**). For both triplexes and G4s, FANCJ requires a 5′ single-stranded DNA tail, consistent with its 5′–3′ directionality of translocation. For unwinding triplexes this 5′ tail must reside on the pyrimidine motif third strand that invades the major groove of the underlying DNA double helix. FANCJ has the capacity to resolve intermolecular (two-stranded or four-stranded) as well as unimolecular G4 substrates (Bharti et al., 2013), which is likely important to suppress replication-associated G4 substrates and in turn DSB formations (discussed below).

**FIGURE 4:**
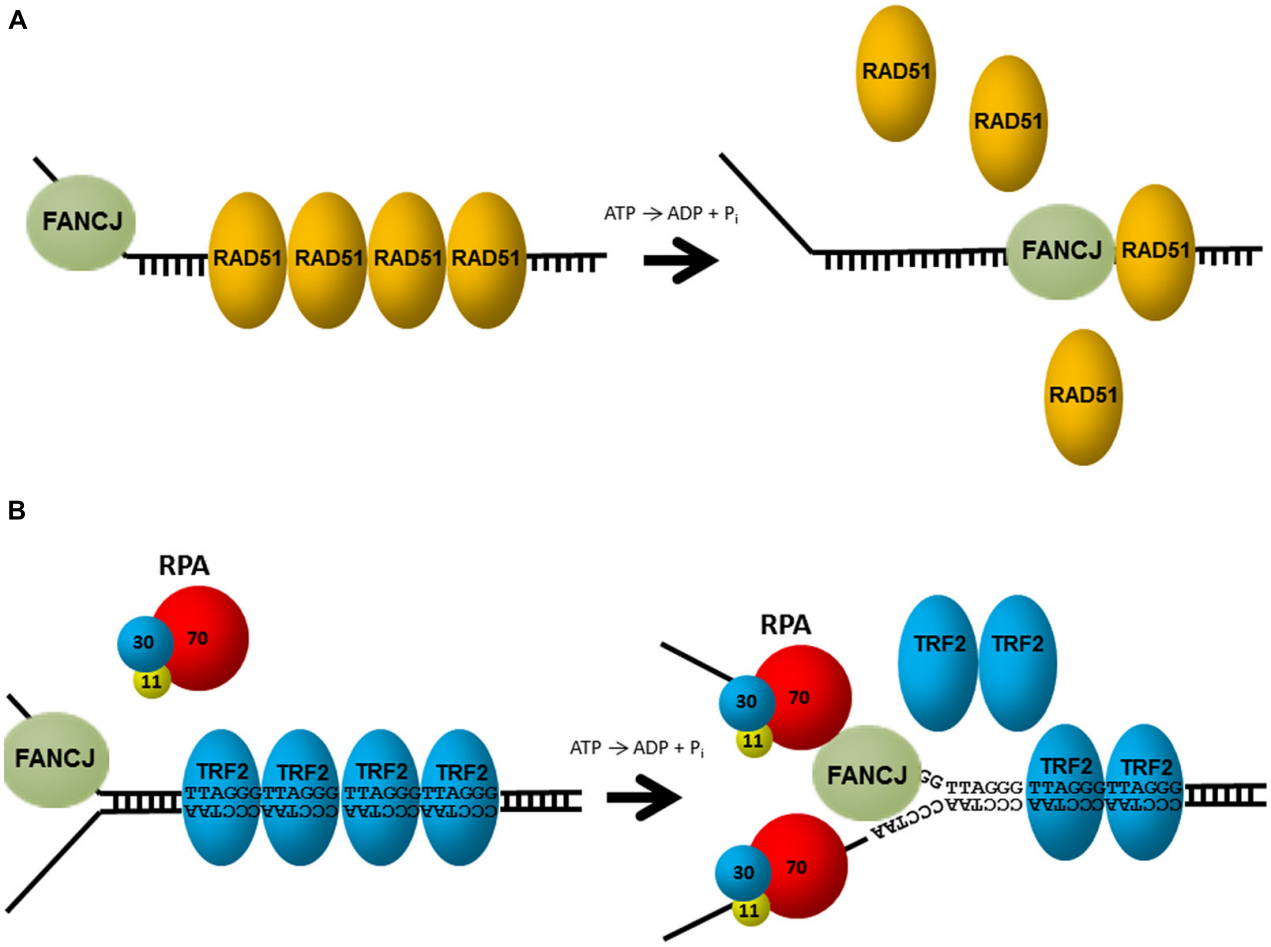
**FANCJ uses its motor ATPase to displace proteins bound to DNA. (A)** FANCJ catalytically displaces the major DNA recombinase protein RAD51. See reference Sommers et al. (2009) and text for details. **(B)** Replication protein A (RPA) stimulates FANCJ to eject the shelterin protein TRF1 (or TRF2) bound to telomeric repeat duplex DNA. See reference Sommers et al. (2014) and text for details.

Aside from unwinding DNA, FANCJ has the ability to harness the energy from ATP hydrolysis to disrupt protein–DNA interactions. Attesting to its robust capacity, FANCJ was shown to disrupt the high affinity interaction of biotin bound to a biotinylated oligonucleotide in an ATP-dependent manner (Sommers et al., 2009). Of greater biological relevance, FANCJ can destabilize a RAD51-single-stranded DNA filament (**Figure 4A**), and therefore inhibit DNA strand exchange activity of RAD51 (Sommers et al., 2009). Thus, FANCJ may limit promiscuous recombination. Alternatively, by removing RAD51 from the 3′ invading strand of the nucleoprotein filament, FANCJ could enable loading of the DNA polymerase and promotes DNA synthesis.

FANCJ helicase and translocase activities are also modulated by protein interactions. In particular, FANCJ is blocked from unwinding partial duplex DNA substrates bound by double stranded DNA-interacting proteins (e.g., catalytically inactive restriction endonuclease, or the telomere binding proteins TRF1, TRF2). However, this inhibition is overcome by the presence of RPA in the FANCJ reaction mixtures under conditions that RPA alone had little effect (Sommers et al., 2014; **Figure 4B**). The ability of RPA to stimulate FANCJ displacement of TRF1 or TRF2 from forked duplex substrates harboring telomeric repeats may be important for remodeling chromosome ends during DNA replication or repair. Indeed, FANCJ was localized to telomeres of living cells that operate according to the alternative lengthening of telomere (ALT) pathway (Dejardin and Kingston, 2009). Since RPA also increases the ability of FANCJ to unwind duplex and G4s (Wu et al., 2008), RPA–FANCJ interactions may both clear protein obstacles and resolve alternate DNA structures during cellular replication to preserve the genome (see below).

Our biochemical studies further suggest that RPA is important for enabling FANCJ to bypass bulky adducts or helix-distorting lesions such as thymine glycol, an oxidative base damage that can be mutagenic or lethal (Wallace, 2002). FANCJ helicase activity is strongly inhibited by a single thymine glycol in either the translocating or non-translocating strand of a DNA duplex (Suhasini et al., 2009). However, RPA stimulates FANCJ to efficiently unwind the substrate harboring thymine glycol in the non-translocating strand, but fails to do so when the thymine glycol resides in the translocating strand. The demonstrated high affinity interaction of RPA with single-stranded DNA harboring a single thymine glycol (Suhasini et al., 2009), together with the strand-specific RPA stimulation of FANCJ helicase activity on the DNA substrate harboring the thymine glycol, suggest a model in which RPA promotes strand displacement. Specifically, the exposed thymine glycol in the non-translocating strand of the partially unwound DNA substrate is readily bound by RPA, resulting in RPA coating of the strand displaced by FANCJ, stabilized duplex separation, and further FANCJ helicase progression past the thymine glycol leading to complete separation of the complementary strands. Such a mechanism may be important for the role of FANCJ to insure timely progression through S phase (Kumaraswamy and Shiekhattar, 2007), or in an environment of heightened oxidative stress. Given the emerging evidence that the FA pathway suppresses DNA damage induced by products of normal cellular metabolism such as aldehydes (Nalepa and Clapp, 2014), it will be of interest to assess how such aldehyde-induced lesions affect FANCJ and its role in DNA repair.

## POST-TRANSLATIONAL MODIFICATIONS OF FANCJ

There has been considerable interest in the effect of post-translational modifications on the functions of DNA repair proteins and checkpoint signaling, both important components of the DNA damage response. Post-translational modifications of DNA damage response proteins can affect their subcellular localization, chromatin association, DNA and protein interactions, stability, and catalytic activity. Phosphorylation of FANCJ mediates interactions promoting repair and checkpoint responses. The first identified was the phosphorylation of Ser-990, which is essential for FANCJ binding to the tandem C-terminal BRCT motifs of BRCA1 (Yu et al., 2003; **Figure 2**). The interface between FANCJ and the interacting BRCA1 BRCT repeats was further defined in structural studies (Yu et al., 2003; Botuyan et al., 2004; Shiozaki et al., 2004). Loss of this Ser-990 phosphorylation limits HR, but also enhances polymerase η dependent bypass suggesting that BRCA1 binding to FANCJ is important for directing the mechanism of DNA damage repair (Xie et al., 2010). Interestingly, the region of FANCJ that binds to the Bloom’s syndrome protein (BLM) overlaps with the FANCJ Ser-990 phosphorylation site (Suhasini et al., 2011; **Figure 2**), raising the possibility that the protein interaction of FANCJ with BLM is affected by phosphoSer-990. More recently, a second FANCJ phosphorylation dependent interaction was identified at Thr-1133 (**Figure 2**). Phosphorylated FANCJ Thr-1133 interacts with the BRCT repeats of Topoisomerase IIb binding protein 1 (TopBP1) to promote an ATR-dependent checkpoint in response to replication stress (Gong et al., 2010).

Acetylation of FANCJ at lysine 1249, the last C-terminal amino acid (**Figure 2**), affects the DNA damage response similar to Ser-990 phosphorylation. When acetylation is prevented, cellular ICL resistance is achieved by a reduced need for Rad54-mediated HR repair and enhanced dependence on the translesion polymerase η. This modulation of repair pathway mechanism could stem from the role of FANCJ and its acetylation upregulating DNA end resection required for HR. Dynamic acetylation of FANCJ was also found to maintain checkpoint signaling following DNA damage (Xie et al., 2012). Continued studies of post-translational modifications on FANCJ and its partners are warranted. For example, it will be important to determine if a post-translational modification of FANCJ or the Bloom’s syndrome helicase (BLM) influences the interaction between the two DNA helicases (**Figure 2**), especially because FANCJ status dramatically influences BLM protein stability by a proteasomal degradation pathway (Suhasini et al., 2011). It remains to be seen if the phosphorylation or acetylation state of FANCJ affects its catalytic activity on a specific DNA substrate. On a forked duplex DNA substrate, acetylation at 1249 did not influence its activity (Xie et al., 2012). Alternatively, post-translational modification of FANCJ may influence its subcellular localization. Recently it was reported that ICL induces localization of FANCJ to the centrosome and FANCJ is involved in regulation of centrosome biogenesis (Zou et al., 2013).

## NOVEL INSIGHTS TO FANCJ STRUCTURE-FUNCTION RELATIONSHIPS BY CHARACTERIZATION OF SITE-DIRECTED MUTANTS

Characterization of helicase missense mutants may be informative for dissecting the molecular basis of disease, or potential dominant negative effects of debilitating missense mutations (Suhasini and Brosh, 2013). For FANCJ, the clinical spectrum of mutations includes missense mutations genetically linked to FA and/or cancer. This is not the general case for a number of disease-causing helicase mutations. For example, only recently were several WRN missense mutations genetically linked to the premature aging disorder Werner syndrome identified and found to be in conserved catalytic domains of the WRN protein (Friedrich et al., 2010). The vast majority of WRN mutations are limited to frameshift or nonsense codons resulting in truncated proteins.

The first FANCJ mutants to be studied were missense variants (P47A, M299I; **Figure 2**) identified in individuals with early breast cancer and normal genotypes for BRCA1 or BRCA2 (Cantor et al., 2001). Tumors of the individuals who carried these two germline FANCJ mutations also carried a copy of the wild-type (WT) allele, suggesting that loss of function may have contributed to the penetrance of the mutant allele by a dominant negative mechanism. Biochemical analysis of the corresponding purified recombinant FANCJ proteins demonstrated that both missense mutations (P47A, M299I; **Figure 2**) affected catalytic activity in a distinct manner. The P47A substitution in the highly conserved Walker A box (motif I) inactivated the ATPase and helicase functions of FANCJ, whereas the M299I mutation located in the Fe-S cluster upregulated its ATPase activity (Cantor et al., 2004). A subsequent study demonstrated that the FANCJ-M299I protein could harness its elevated ATP hydrolysis to unwind a DNA substrate with damage in its sugar phosphate backbone in a more proficient manner (Gupta et al., 2006). Based on these studies, it was proposed that perturbation of FANCJ catalytic activity interferes with the helicase’s normal role in the DNA damage response leading to tumorigenesis; however, a better understanding of FANCJ’s precise role in cellular transformation is required. As discussed below, expression of certain FANCJ mutant proteins in a normal FANCJ background confer sensitivity to DNA damaging agents, suggesting that a single mutant FANCJ allele could be pathogenic. Further characterization of FANCJ variants using cell- and animal-based models may be helpful.

Analysis of the FA-associated FANCJ-A349P mutant provided insight to the pathogenesis of FA and the role of the Fe-S domain (Wu et al., 2010; **Figure 2**). The alanine to proline substitution is adjacent to one of the highly conserved cysteine residues important for chelation of Fe atoms. Inheritance of the A349P mutation and a second mutation encoding a prematurely truncated FANCJ protein resulted in intrauterine growth failure and death as a stillborn fetus with a gestational age of 22 weeks (Levran et al., 2005). Our genetic analysis demonstrated that expression of the FANCJ-A349P mutant allele in FANCJ-null FA-J patient cells failed to rescue sensitivity to the DNA cross-linking agent MMC; similarly, expression of FANCJ-A349P in fancj null chicken DT40 cells failed to rescue cisplatin sensitivity (Wu et al., 2010). Moreover, expression of the FANCJ-A349P mutant in FA-J cells or fancj null chicken cells failed to restore resistance to the G4 ligand telomestatin (TMS). These studies suggested that the FANCJ-A349P mutant is defective for ICL processing and G4 unwinding *in vivo*. Moreover, expression of FANCJ-A349P in cells expressing the normal FANCJ protein exerted a dominant negative effect, presenting the possibility that a single mutant FANCJ allele could be pathogenic. *In vitro* analysis revealed that the A349P substitution interfered with the functionality of the FANCJ Fe–S cluster and uncoupled ATP-dependent DNA translocation from helicase activity on duplex or G4 DNA substrates (Wu et al., 2010). Thus, an intact Fe–S domain is critical for FANCJ DNA unwinding and this activity is fundamentally important for FANCJ ICL repair and G4 DNA metabolism (Wu et al., 2010). It remains to be determined if the functions of FANCJ in ICL repair and G4 unwinding are linked or if separation-of-function mutants exist. Ongoing efforts in this area could elucidate the importance of FANCJ in the FA pathway of cross-link repair versus functions outside the FA pathway that may also be significant for genome stability.

FANCJ also has a conserved Q motif (also called motif 0) found in both RNA and DNA helicases that is predicted to coordinate ATP binding and hydrolysis to catalytic DNA strand separation (Tanner et al., 2003; **Figure 2**). Biophysical analysis of the purified recombinant FANCJ-Q25A mutant protein disrupted in the Q motif revealed that its assembly state was dramatically altered compared to the recombinant WT FANCJ protein. FANCJ-Q25A protein was only a monomer, whereas FANCJ-WT protein was nearly equally monomer and dimer (Wu et al., 2012). Thus, the Q motif in FANCJ plays a critical role in multimerization. FANCJ-Q25A was defective for DNA binding, ATP hydrolysis, and helicase activity. Consistent with the biochemical results, expression of the FANCJ-Q25A mutant protein in fancj null cells failed to rescue their sensitivity to a DNA cross-linking agent or G4 ligand. Moreover, expression of the FANCJ-Q25A mutant allele in a WT FANCJ background resulted in dominant negative phenotypes for ICL or G4 ligand resistance. Co-immunoprecipitation experiments with nuclear extracts demonstrated that the FANCJ-Q25A mutant protein retained its ability to interact with known protein partners of FANCJ (TopBP1, BRCA1), suggesting that these retained interactions could contribute to the dominant negative nature of the mutant allele; however, further studies are required to ascertain the precise mechanism. From a clinical perspective, it is of significance that the FANCJ-Q25A mutant is similar to a patient-derived BLM missense mutation in the Q motif (Q672R) that impairs BLM foci formation after cellular exposure to agents that impose replication stress (Ellis et al., 1995; Wu et al., 2012). Therefore, dimer formation may be essential for FANCJ focal accumulation and function *in vivo*.

Two FA patient-derived missense mutations in motif Ia, R251C, and Q255H, were characterized by the Wu lab (Guo et al., 2014; **Figure 2**). Although expression of either R251C or Q255H mutant could not rescue the cisplatin sensitivity of a fancj null cell line, the two mutations exerted markedly different effects on the biochemical functions of FANCJ *in vitro*. Both FANCJ motif Ia mutants abolished DNA helicase activity. The R251C mutation strongly interfered with FANCJ DNA binding and consequently its DNA-dependent ATPase activity. Instead, the Q255H mutant displayed elevated FANCJ DNA binding, a normal ATPase function, and ability to translocate on single-stranded DNA. In this regard, the Q255H mutant behaved similarly to the FANCJ-A349P mutant which was also able to translocate on single-stranded DNA in an ATP-dependent manner but failed to unwind even short 20 bp duplex substrates (Wu et al., 2010). For either the Q255H or R251C mutant, the ability to translocate on single-stranded DNA did not translate into efficient disruption of protein-DNA complexes. Collectively, these studies suggest that FANCJ translocase activity without protein complex disruption and/or DNA unwinding is insufficient for its *in vivo* function. Nevertheless, the dominant negative phenotype exerted by FANCJ mutant alleles (A349P, Q25A, R251C, and Q255H) that impair DNA helicase (but not necessarily ATPase activity) demonstrate that catalytic DNA unwinding is vital for FANCJ function and suggests that FANCJ heterozygosity may contribute to tumorigenicity or disease-associated phenotypes. For a further discussion of helicase-inactivating mutations as a basis for dominant negative phenotypes, see (Wu and Brosh, 2010b).

Evidence continues to build that FANCJ and other players in the FA pathway are *bona fide* tumor suppressor genes, even outside their roles in the FA pathway (Pickering et al., 2013; Park et al., 2014; Pauty et al., 2014). For example, whole genome-sequencing of Icelanders led researchers to discover frameshift mutations in the BRIP1 (FANCJ) gene that vastly elevate the risk of invasive ovarian cancer (Rafnar et al., 2011). Moreover, a BRIP1 frameshift mutation was associated with a 36% increased risk of cancer in general and a reduced lifespan of 3.6 years compared to non-carriers (Rafnar et al., 2011). This work and that of others, reviewed in (Cantor and Guillemette, 2011), emphasizes the prominent role of FANCJ as a tumor suppressor, which may be informative for future studies in personalized medicine that exploit the mutational status of FANCJ and other DNA repair helicases (see below). The spectrum of associated cancers could be broad. Indeed, we found a number of protein coding mutations in FANCJ in melanoma genomes (one allele), suggesting that FANCJ deficiency may be a risk factor for skin cancer and possibly associated tumors could be sensitive to ICL-inducing agents (Guillemette et al., 2014).

## FANCJ AND ITS PROTEIN PARTNERS ARE RECRUITED TO DNA DAMAGE FOCI IN A REGULATED AND LESION-SPECIFIC MANNER

Given the direct interaction of FANCJ with BRCA1, the dependency of these proteins on each other for localization at lesions has been examined. Indeed, these proteins colocalize at sites of DNA damage foci following HU, IR, and at laser-induced stripes (Cantor et al., 2001; Greenberg et al., 2006). BRCA1 mutant cells display reduced immunofluorescent focal staining for FANCJ in untreated cells as well as cells exposed to DNA damaging agents (Cantor et al., 2001; Gupta et al., 2007). However, the contribution of FANCJ to BRCA1 localization to DNA damage sites may be time or context dependent. FANCJ status did not affect recruitment of BRCA1 to laser-induced DSBs or psoralen (Pso)-ICLs (Suhasini et al., 2013). However, the number and intensity of BRCA1 foci in FANCJ-deficient cells exposed to IR was reduced at time points as early as 1 h post irradiation (Peng et al., 2006). Thus, there may be separate pools of FANCJ and BRCA1 or a sub-fraction of FANCJ may contribute to BRCA1 recruitment or retention at foci after IR exposure. Controlling the localization of BRCA1 to sites of DNA damage would directly affect its DNA repair function, and have potential consequences for cellular homeostasis. For example, oncogenic RAS transformation down-regulates FANCJ expression, which causes BRCA1 dissociation from chromatin, resulting in an impaired DNA damage response leading to cellular senescence (Tu et al., 2011).

**FIGURE 5:**
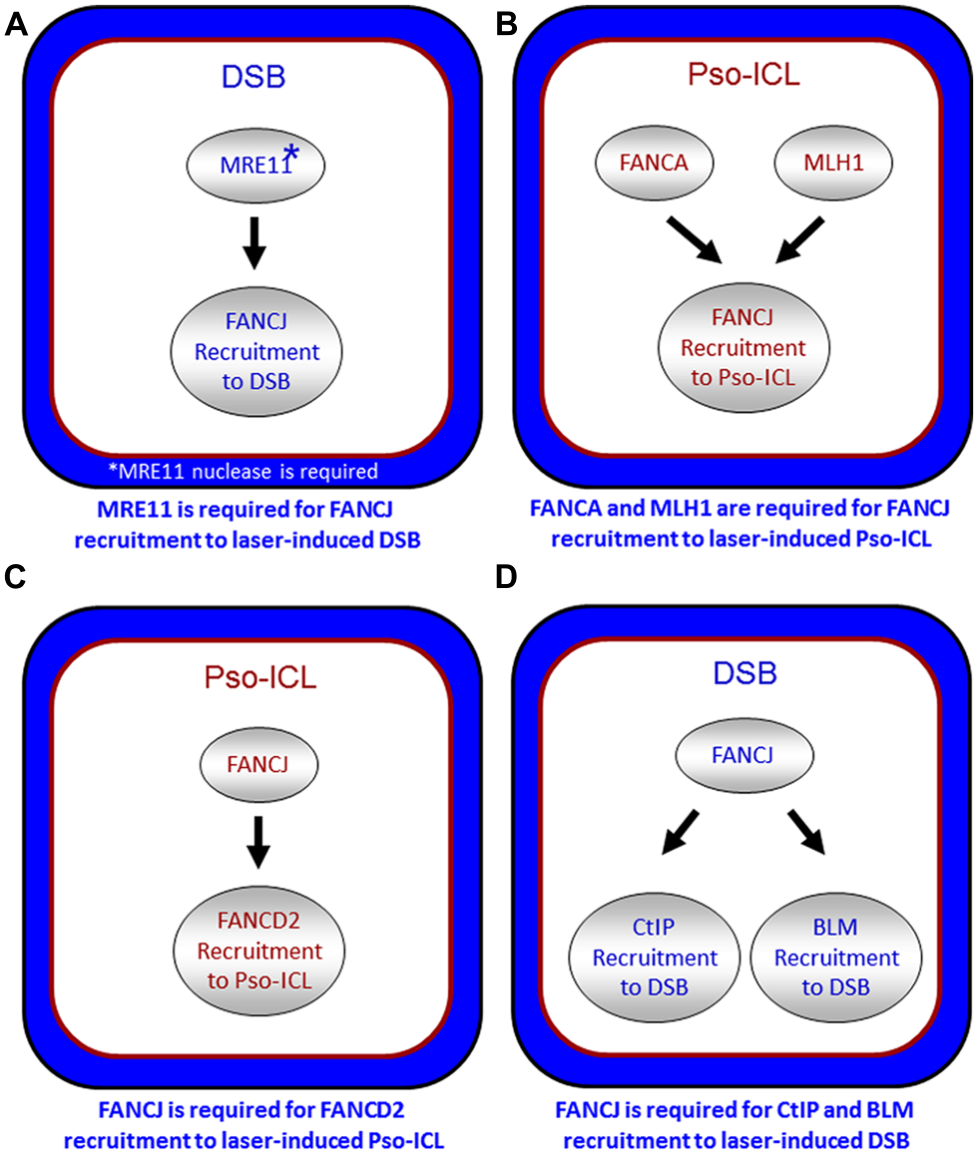
**Spatio-temporal recruitment of FANCJ and its protein partners to site of DNA damage.** Confocal microscopy studies with living cells were used to elucidate the requirements of FANCJ or its interacting DNA repair factors to laser-induced psoralen (Pso)-interstrand cross-links **(A,C)** or laser-induced double strand breaks (DSB; **B,D**). See reference Suhasini et al. (2013) and text for details.

It is also important to note that other DNA repair proteins function with or in parallel with BRCA1 localize FANCJ to sites of DNA breaks. In particular, the DSB repair protein MRE11 and its associated nuclease activity is necessary for efficient FANCJ recruitment to laser-induced DSBs (Suhasini et al., 2013; **Figure 5A**). Consistent with this observation, CtIP is also delayed in its recruitment to DSBs in cells that are deficient in MRE11 exonuclease, that have reduced FANCJ recruitment to DSBs (Suhasini et al., 2013). Recent work from the Paull laboratory has implicated a catalytic role of CtIP in 5′ strand resection that is involved in the removal of DNA adducts at DNA breaks (Makharashvili et al., 2014). Perhaps FANCJ collaborates with CtIP to remove secondary structures therefore enabling CtIP to efficiently incise and process DNA ends, such as at common fragile sites (Wang et al., 2014). It is plausible that FANCJ’s role in end resection may be independent of BRCA1, similar to what was determined for CtIP (Polato et al., 2014).

A critical protein partner that FANCJ collaborates with is RPA (Gupta et al., 2007). FANCJ and RPA robustly co-localize after DNA damage induced by IR or MMC or replication stress imposed by hydroxyurea (HU), which depletes nucleotide pools. RPA foci formation was not dependent on FANCJ mutational status after MMC treatment or IR exposure (Gupta et al., 2007), suggesting that FANCJ helicase activity is not a prerequisite for creating single-stranded DNA at DNA breaks that RPA nucleates on. However, RPA foci are reduced in FANCJ-deficient cells exposed to HU for a short time period (20 min; Gong et al., 2010) or ultraviolet (UV) light (Guillemette et al., 2014), suggesting FANCJ helicase activity provides single-stranded DNA loading zones for RPA when the replication fork is stalled. RPA binds to FANCJ and stimulates its helicase (Gupta et al., 2007) and DNA-protein displacement activities (Sommers et al., 2014), as mentioned above. Based on these observations, we favor the hypothesis that FANCJ together with RPA binds a key DSB repair intermediate or stalled replication fork structure and RPA stimulates FANCJ’s helicase and/or protein displacement activity to allow appropriate and efficient processing during the maturation of the DNA intermediate.

FANCJ also binds directly to the mismatch repair (MMR) protein MLH1 (Peng et al., 2007). While MLH1 binding did not demonstrate any notable changes in FANCJ helicase activity *in vitro*, MLH1 is critical for FANCJ localization to sites of DNA crosslinks. In particular, using a laser confocal microscopy approach with living cells, we examined the recruitment of FANCJ to laser-activated Pso-ICLs (Suhasini et al., 2013). This analysis demonstrated that FANCJ relies on MLH1 and a member of the FA core complex, FANCA, to recruit efficiently to the laser-activated Pso-ICL (**Figure 5B**). FANCJ localization to UV light induced DNA crosslinks also requires the MLH1 interaction as well as the upstream MMR protein MSH2 (Guillemette et al., 2014). This is logical given that the physical interaction between FANCJ and MLH1 is required for cells to properly respond to agents that induce ICL damage or UV damage (Peng et al., 2007; Guillemette et al., 2014). Localization of FANCJ by MMR proteins may in turn limit deleterious MMR functions at a stalled fork. This idea is based on the fact that defects in cells lacking the FANCJ-MLH1 interaction are suppressed by depletion of MSH2 (Peng et al., 2014). Coordination by FANCA and MMR proteins could ensure that FANCJ helicase function is set to unwind DNA or displace proteins to restore replication fork progression following ICLs or other replication blocking agents.

The relationship of FANCJ with FANCD2 is more complex. While FANCJ operates downstream of FANCD2 monoubiquitination (Litman et al., 2005; **Figure 1**), FANCD2 recruitment to Pso-ICLs, not but laser-induced DSBs, was dependent on FANCJ (Suhasini et al., 2013; **Figure 5C**). Consistent with these observations, FANCJ is recruited to Pso-ICLs much earlier than FANCD2. These findings correlate with those of the Andreassen lab that reported that FANCJ foci formed normally in FANCD2-deficient cells after exposure to the DNA cross-linker MMC (Zhang et al., 2010). In more recent studies, the Kupfer lab reported that FANCD2 is required for proper chromatin localization of FANCJ, suggesting a mechanism whereby FANCD2 helps to regulate FANCJ’s role in downstream events of the FA pathway (Chen et al., 2014). In addition, they propose a model in which FANCJ sequesters non-monoubiquitinated FANCD2 from chromatin in the absence of DNA damage; therefore, a collaborative interaction between FANCJ and FANCD2 could exist that is necessary for the appropriate DNA damage-induced chromatin association of the two FA proteins.

FANCJ also binds and serves to localize the BLM helicase to DSBs (Suhasini et al., 2013; **Figure 5D**). The precise role of FANCJ in BLM recruitment to laser-induced DSBs or its function at DNA ends is being investigated, and a potential partnership between FANCJ and BLM in processive strand resection is a possibility (Suhasini and Brosh, 2012). Based on biochemical evidence that FANCJ and BLM helicases synergistically unwind damaged DNA (Suhasini et al., 2011), we proposed a model that the two helicases with opposite directionalities of translocation move together in a complex as part of the end resection machinery involving RPA and the 5′ structure-specific nucleases DNA2 or EXO-1 to catalytically resect single-stranded DNA to provide the 3′ single-stranded overhang for strand invasion step of HR repair (Suhasini and Brosh, 2012). Our recent work showing that FANCJ or a human RecQ helicase (RECQ1) can efficiently dislodge protein bound to duplex DNA in a RPA-dependent manner (Sommers et al., 2014) poses a scenario in which FANCJ and BLM with their interacting partner RPA displace proteins bound near double-stranded ends and resolve secondary structure or damaged DNA to enable processive and kinetically efficient end resection.

Clearly a concerted hierarchy exists for FANCJ and its interacting partners to be recruited to DNA damage sites and subsequently act. Further studies are needed because they may provide insights to DNA repair pathway cascades or the cross-talk between DNA damage response regimes. For example, the interaction of FANCJ with the MRN complex and BLM helicase suggests that FANCJ may have both early and late roles in DSB repair. FANCJ has the ability to inhibit MRE11 3′–5′ exonuclease activity (Suhasini et al., 2013), which may serve to harness initial end trimming by MRE11 to avoid excessive end resection that would generate 5′ single-stranded tailed duplex. Secondly FANCJ with RPA may facilitate processive strand resection by its interaction with the BLM-DNA2 or BLM-EXO-1 complexes to yield the 3′ single-stranded tailed duplex. Biochemical reconstitution experiments with purified proteins and defined DNA substrates, as well as carefully designed cell-based assays should address the efficacy of these models.

**FIGURE 6:**
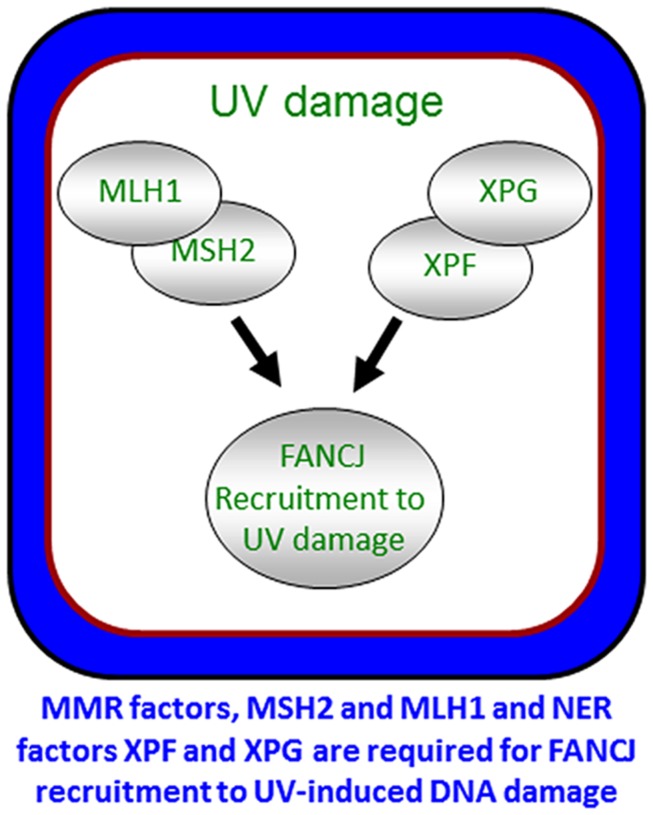
**Localization of FANCJ to UV-induced DNA damage.** FANCJ recruitment to UV-induced DNA damage is dependent on the mismatch repair factors MLH1 and MSH2, and NER factors XPF and XPG. See reference Guillemette et al. (2014) and text for details.

To ensure a robust DNA damage response and coordinate repair processing, it appears that more than one pathway contributes to the localization of FANCJ. As illustrated above, FANCJ recruitment to ICLs or DSBs is determined by proteins that either interact directly with FANCJ or operate in the same pathway. Indeed, both nucleotide excision repair (NER) and MMR proteins promote the localization of the FANCJ to sites of UV-induced lesions (Guillemette et al., 2014; **Figure 6**). MMR proteins initially recruit FANCJ. However, the further accumulation of FANCJ requires dual incision by the NER endonucleases XPF and XPG (Guillemette et al., 2014). Conceivably, the NER-dependent incision provides an ideal substrate for FANCJ at the lesion site. The combined MMR and NER localization of FANCJ ensures an S-phase checkpoint, lesion repair, and the suppression of UV-induced mutations. Supporting that multiple pathways contribute to high fidelity repair after UV irradiation, similar to skin tumors from XP patients (Dumaz et al., 1993), MMR-deficient (Borgdorff et al., 2006) and FANCJ-deficient (Guillemette et al., 2014) cells display an elevated frequency of UV-induced C > T point mutations. Moreover, along with NER genes, FANCJ and MMR genes are mutated in melanoma (Guillemette et al., 2014). The NER and FA-associated XPF protein promotes RPA phosphorylation in S-phase cells (Bomgarden et al., 2006). Given that XPF promotes FANCJ accumulation in S-phase cells, it follows that FANCJ also functions to promote RPA phosphorylation throughout S-phase. This function could be shared by FANCJ partners, such as BLM or the FA pathway, explaining its link to the UV response and checkpoints that limit genomic instability (Suhasini et al., 2011; Kelsall et al., 2012; Nalepa et al., 2013; Singh et al., 2013).

## ROLE OF FANCJ AND THE FA PATHWAY IN THE BROADER CONTEXT BEYOND DNA REPAIR

Much emphasis has been placed on the impact of ICLs on actively dividing cells and role of the FA pathway in repairing such lesions encountered by the replication for. ICLs may arise naturally or be induced exogenously by chemicals used in chemotherapy. It is important to note that ICLs may have detrimental effects in non-dividing cells, given that they would interfere with other processes such as transcription. This has become a topic of increased interest, particularly because the processes of DNA repair and transcription are both highly important and inter-related. Human diseases with inherited defects in classic DNA repair genes (e.g., nucleotide excision repair) often display transcriptional deficiencies which are likely to culminate in pleiotropic symptoms including developmental abnormalities and features of premature aging [for review, see (Kamileri et al., 2012a)]. For example, the XPF-ERCC1 nuclease responsible for ICL unhooking in the FA pathway is recruited to active promoters and implicated in chromatin modifications that influence transcriptional activation (Le May et al., 2010; Kamileri et al., 2012b). Alterations to chromatin packaging due to mutations in DNA repair genes are suspected to underlie the phenotypic defects that contribute to developmental disorders that extend beyond DNA repair and maintenance of genomic stability. The connection of ICL accumulation to perturbed transcriptional regulation in an ERCC1- defective model of a human progeroid syndrome characterized by loss of fat tissue suggests how DNA damage can inflict detrimental effects in non-dividing cells (Karakasilioti et al., 2013). Defects in the FA pathway fall into this class of diseases as the clinical symptoms include not only cancer and hematologic abnormalities but often a range of congenital issues that can include skeletal defects and short stature, as well as renal dysfunction, abnormal pigmentation, and osteoporosis (Kee and D’Andrea, 2012). As mentioned above, FANCD2 is proposed to control FANCJ’s localization to chromatin and its involvement in downstream events of the FA pathway (Chen et al., 2014). Therefore, it is of great importance to ascertain the probable dependent and independent roles of FANCJ and the FA pathway in transcriptional regulation. In terms of FANCJ, a leading hypothesis builds from its role as a G4 resolving enzyme that may target predicted G-quadruplexes (G4s) found near promoter elements believed to regulate transcription initiation (see below).

## INVOLVEMENT OF FANCJ DURING REPLICATION STRESS

Replication stress can be a source of genomic instability. Cellular data have implicated FANCJ in helping to cope with replication stress induced exogenously by chemical exposure. Human cells mutated in FANCJ or acutely depleted of FANCJ by RNA interference are sensitive to DNA cross-linking agents that block fork progression (Bridge et al., 2005; Litman et al., 2005; Peng et al., 2007), or HU that causes fork stalling (Suhasini et al., 2011). Even in the absence of agents that exogenously induce replication, a role for FANCJ helicase activity to insure timely progression through S phase has been demonstrated (Kumaraswamy and Shiekhattar, 2007). FANCJ is implicated in intra-S phase checkpoint signaling through its interaction with TopBP1, which allows for activation of ataxia telangiectasia and Rad3-related (ATR), a requirement for checkpoint kinase 1 (CHK1; Gong et al., 2010). However, it is still unclear what the function(s) of FANCJ may be to influence events associated with fork stalling. When the replisome encounters a replication-blocking lesion or stalls due to replication stress induced by a small molecule that impedes the replicative helicase or depletes the nucleotide pool, the nascent leading and lagging strands can anneal to each other to form a Holliday Junction-like chicken foot DNA structure [for review, see (Atkinson and McGlynn, 2009)]. This process is known as fork regression or fork reversal, and provides a mechanism for repair machinery to gain access to the lesion as well as stabilize the fork. Whereas certain RecQ helicases (e.g., WRN, BLM; Machwe et al., 2006; Ralf et al., 2006) were shown to support fork regression, FANCJ is not directly implicated in this process. However, as a 5′–3′ DNA helicase, FANCJ may collaborate with a 3′–5′ helicase (e.g., WRN, BLM) to promote fork regression. Given that FANCJ has already been demonstrated to interact with BLM (Suhasini et al., 2011), the FANCJ–BLM partnership may contribute to concerted fork regression by opposite polarity helicases.

Aside from its proposed role in DNA end processing to initiate HR repair (previous section), FANCJ may help to process DNA structures associated with stalled replication forks or regulate such processing events. The interaction between FANCJ and MLH1 appears to be critical for cells to recover from replication stress induced by ICLs or the DNA polymerase inhibitor, aphidicolin. As mentioned above, deletion of MSH2 suppresses replication restart defects in cells lacking the FANCJ–MLH1 interaction (Peng et al., 2014). Thus, to restart stalled replication forks, FANCJ through its MLH1 interaction could normally unwind and eliminate DNA structures bound by MSH2 or displace MSH2 from such structures. Mre11 has been implicated in the restart of stalled replication forks (Bryant et al., 2009; Hashimoto et al., 2010). As proposed for the tumor suppressor and HR factor BRCA2 as well as the DNA damage sensor poly (ADP-ribose) polymerase 1 (PARP1; Schlacher et al., 2011; Ying et al., 2012), FANCJ may help to prevent uncontrolled MRE11-dependent degradation of stalled replication forks by inhibiting its nuclease activity. Such an anti-nuclease role may aid in fork stabilization without excessive single-stranded DNA production to allow for regulated checkpoint signal transduction and effective repair of the replication-blocking lesion.

## FANCJ RESOLVES G-QUADRUPLEX DNA TO ENABLE SMOOTH REPLICATION AND PRESERVE GENOMIC INTEGRITY

**FIGURE 7:**
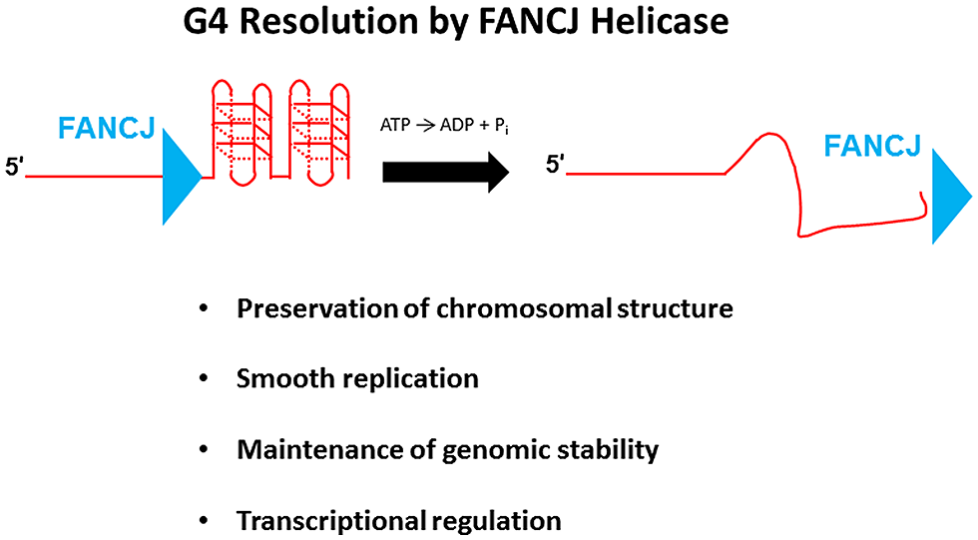
**FANCJ resolves G-quadruplex DNA to maintain genomic stability and cellular homeostasis.** See text for details.

G-quadruplexes, composed of planar stacks of four guanine residues engaged in Hoogsteen hydrogen bonding, are now believed to form *in vivo* and exert biological effects on DNA replication and transcription, and play unique structural roles in telomere capping (Wu and Brosh, 2010a; Bochman et al., 2012; Maizels and Gray, 2013). FANCJ, and certain other DNA helicases (e.g., PIF1, WRN, BLM), resolve G4 DNA structures *in vitro*; however, the biological significance of G4 resolution by the various human DNA helicases *in vivo* is less clear. Human cells deficient in FANCJ, but not FANCA or FANCD2, were found to be sensitive to the G4 binding drug TMS, suggesting a role of the helicase to preserve genomic stability at G4-forming sequences that is independent of the classic FA DNA repair pathway (Wu et al., 2008); furthermore, a FA-J human patient cell line was found to accumulate deletions at predicted G4-forming sequences in the genome (London et al., 2008). Although a number of DNA helicases have been shown to unwind intermolecular G4 structures, FANCJ is distinct among the Fe-S cluster helicases tested in its ability to resolve entropically favored intramolecular G4 substrates (Bharti et al., 2013). Consistent with the biochemical results, a deficiency in FANCJ, but not the Fe–S helicases DDX11 or XPD, sensitized human cells to TMS as measured by induction of the DNA damage marker γ-H2AX (Bharti et al., 2013). These findings suggest that FANCJ has a specialized function among the Fe–S helicases to facilitate smooth replication of genomic regions prone to form G4s in order to prevent fork breakage (**Figure 7**). A recent study using Xenopus egg extract and single-stranded plasmid DNA template with a predicted G4-forming sequence validated the role of FANCJ to promote DNA synthesis through G4 structures (Castillo et al., 2014). It will be of interest to determine if a putative role of FANCJ at telomeres involves its ability to resolve the G-rich telomeric tail and influence telomere capping events.

Recent advances using the chicken DT40 cell lines have improved our understanding of the role of FANCJ in DNA repair and G4 DNA metabolism. Human FANCJ can rescue the sensitivity of chicken brip1/fancj mutant cells to agents that induce DNA cross-links (Bridge et al., 2005) or stabilize G4 structures (Wu et al., 2008), indicating a conservation of function in the vertebrate species. In addition to its role in protecting brip1 cells from G4-associated genomic instability, the chicken ortholog of FANCJ serves a more general protective role in chromosomal maintenance that appears to operate outside the FA pathway (Kitao et al., 2011; **Figure 7**). In subsequent work, it was shown that FANCJ, together with the RecQ helicases WRN and BLM, preserve epigenetic stability by helping to efficiently couple histone recycling with replication fork progression through their ability to resolve G4s that impede smooth DNA synthesis (Sarkies et al., 2012). FANCJ seems to be a central player in the maintenance of epigenetic stability by collaborating as well with the REV1 translesion polymerase at G-rich sequences predicted to form quadruplexes. The precise molecular mechanisms of how these processes occur are still not well understood, but the model proposed by the Sale group predicts that FANCJ with its opposite polarity of directional movement may initiate its action on the opposite side of G4 structure(s) as that of the 3′–5′ RecQ heli or the TLS polymerase REV1 (Sarkies et al., 2012).

To examine the role of FANCJ in cellular DNA replication, the Niedzwiedz lab employed a DNA fiber analysis in isogenic fancj null and WT DT40 cells and determined that FANCJ helps to promote replication fork progression at regions prone to stalling, such as G4-forming sequences (Schwab et al., 2013). Furthermore, stabilization of G4 structures in FANCJ-deficient cells by the G4 binder TMS quenched fork progression, resulting in uncoupling of leading and lagging strand synthesis. The ability of FANCJ to enable smooth replication fork progression would help to maintain normal chromatin structure by preventing its condensation and reorganization.

FANCJ-deficient DT40 cells exposed to the G4 ligand TMS displayed stronger staining by a murine monoclonal antibody specific for binding G4 DNA compared to untreated fancj null cells, or normal cells exposed to TMS (Henderson et al., 2013). Enrichment of G4 DNA in FANCJ-deficient cells exposed to TMS provided the first evidence that genetic and environmental conditions can synergize in the accumulation of G4 DNA *in vivo*. The emerging evidence for a role of FANCJ in replication of G4 motifs in avian cells supports the hypothesis for a conserved role of FANCJ in humans. Nonetheless, there are likely additional functions of FANCJ G4 resolving activity *in vivo*, including the control of gene expression (**Figure 7**); however, this hypothesis remains to be formally tested in human cells. It is well known that G-rich sequences predicted to form G4 are enriched in promoter regions especially at transcriptional start sites (Huppert and Balasubramanian, 2007), and a correlation exists between the presence of predicted G4 and promoter-proximal transcriptional pausing (Eddy et al., 2011). Although a definitive role of G4 resolution by FANCJ to control gene expression has not yet been elucidated, other DNA helicases including XPB, XPD (Gray et al., 2014), BLM (Nguyen et al., 2014), and RECQ1 (Li et al., 2014) bind G4 structures and regulate expression of genes characterized by the presence of G4 DNA motifs. It is unclear if differential roles in transcriptional regulation exist for G4 binding versus resolution by a DNA helicase, given that certain helicases [e.g., RECQ1 (Popuri et al., 2008; Wu et al., 2008) and XPB (Gray et al., 2014)] bind but poorly unwind G4 DNA *in vitro*; furthermore, it is yet unclear if human XPD resolves G4 DNA because different results were obtained from *in vitro* studies using XPD proteins from two distinct thermophilic species (Bharti et al., 2013; Gray et al., 2014). Given the evidence that FANCJ and its homologs play an important role in G4 metabolism, it seems likely that FANCJ unwinding of G4s will influence gene expression of proto-oncogenes where G4 motifs are prominent (Eddy and Maizels, 2006; Duquette et al., 2007; Brown et al., 2011). Further studies are needed to establish direct links and meaningful relationships between disease pathogenesis and regulated expression of messenger RNA and microRNA molecules by FANCJ and other DNA helicases.

## FANCJ AS A POTENTIAL TARGET FOR CLINICAL AND PHARMACEUTICAL TREATMENT

Our group and others have been keenly interested in the prospect of helicase-based biomarkers and targeting DNA helicases like FANCJ to enhance existing or developing therapeutic strategies for treating cancers (Brosh, 2013). This prospect has been fueled by observations that the expression of many DNA damage response genes is up-regulated in rapidly proliferating cells and tumors, leading to their resistance to chemotherapy drugs or radiation used to combat cancer. In two recent studies, the influence of FANCJ expression on sensitivity of cancer cells or tissues to chemotherapy drugs was determined. In the first, Nakanishi et al. (2012) found that FANCJ expression in tumor tissues was elevated compared to normal epithelial tissue, which correlated with resistance to the chemotherapy drug 5-fluorouracil (5-FU) in tumors with normal MLH1 expression. Mori et al. (2013) determined that gastric cancer cells exposed to 5-FU down-regulated FANCJ expression, leading to their enhanced sensitivity to the ICL-inducing agent oxaliplatin. Such observations raise the exciting possibility that FANCJ is differentially expressed in tumors which may serve as a useful predictive biomarker for designing treatment strategies tailored to the cancer type.

From a related perspective, the development of small molecules that target FANCJ for helicase inhibition may provide a means to achieve synthetic lethality with chemotherapy drugs or in a defined genetic background to kill cancer cells, provided that a therapeutic window is achieved. Our work on the discovery of a WRN helicase inhibitor provided a proof-of-principle for the helicase-targeted approach to induce chemical or genetic synthetic lethality of cancer cells (Aggarwal et al., 2011, 2013a,b). The observation that a BLM helicase inhibitor can cause elevated sister chromatid exchange in cultured cells is provocative (Nguyen et al., 2013), and sets the stage to search for FANCJ inhibitors, given FANCJ’s interaction with BLM and the finding that acute depletion of FANCJ causes elevated sister chromatid exchange (Suhasini et al., 2011). On the opposite side of the spectrum, it might also be useful to conduct small molecule screens to identify compounds that restore the function of a misfolded helicase protein caused by a disease-linked mutation. Given that a number of missense mutations in FANCJ are linked to FA or associated with cancer, FANCJ may be a good candidate protein to target for intervention.

Lastly, the function of FANCJ in G4 DNA metabolism suggests an avenue to explore. G4 structures which form at telomeres or the promoters of proto-oncogenes most likely play a prominent role in the ability of cancer cells to thrive or senesce. Indeed, telomerase inhibitors which prevent the elongation of the G-rich telomere tails at chromosome ends are in clinical trials (Buseman et al., 2012). If G4 structures in cancer cells are stabilized by G4 ligands directly or by blocking telomeric G4 unwinding with small molecules against G4 resolving helicases such as FANCJ or PIF1, then cancer cells may be caused to senesce and ultimately targeted for elimination. It seems probable that further studies will address the efficacy of targeting FANCJ and other DNA helicases as a possible strategy in cancer therapy.

## SUMMARY

In this review, we have attempted to provide a comprehensive view of what now appears to be multi-faceted roles of FANCJ in cellular DNA metabolism. Discovered 13 years ago as a BRCA1-interacting protein, FANCJ helicase is now regarded as a *bona fide* tumor suppressor and genetically linked to the cancer-prone disorder FA characterized by progressive bone marrow failure; however, the precise function(s) of FANCJ in the FA pathway of ICL repair is still not well understood. In addition, cellular and biochemical studies have demonstrated an important role of FANCJ to resolve G4 DNA structures that potentially interfere with normal chromosome packaging, replication, and transcription. FANCJ interacts with a number of DNA damage response proteins and appears to be a key player in maintaining chromosomal stability through its involvement in DNA repair and checkpoint signaling. With FANCJ’s emergence as a uniquely important genome caretaker, future studies may explore FANCJ as a potential target for clinical and therapeutic strategies. In spite of the tremendous progress, much still remains to be learned about FANCJ and avenues for biomedical advances.

## Conflict of Interest Statement

The authors declare that the research was conducted in the absence of any commercial or financial relationships that could be construed as a potential conflict of interest.
